# Elevated Resting Heart Rate in Hospitalized Patients With Atrial Fibrillation Is Associated With Increased Cardiovascular Risk

**DOI:** 10.1155/cdr/2106637

**Published:** 2026-01-26

**Authors:** Kangning Han, Xia Li, Biao Fu, Mengmeng Li, Tong Liu, Ying Peng, Jing Liu, Na Yang, Yongchen Hao, Chenxi Jiang, Ribo Tang, Jianzeng Dong, Dong Zhao, Deyong Long, Changsheng Ma

**Affiliations:** ^1^ Department of Cardiology, Beijing Anzhen Hospital Affiliated to Capital Medical University, Beijing, China, anzhen.org; ^2^ Department of Mathematical and Physical Sciences, La Trobe University, Melbourne, Australia, latrobe.edu.au; ^3^ Department of Epidemiology, Beijing Anzhen Hospital Affiliated to Capital Medical University, Beijing, China, anzhen.org

**Keywords:** all-cause mortality, atrial fibrillation, heart failure, heart rate control, resting heart rate

## Abstract

**Background:**

Effective rate control is important in the management of atrial fibrillation (AF). However, the relationship between resting heart rate (RHR) and adverse outcomes in hospitalized patients remains uncertain.

**Objective:**

This study was to evaluate the association between RHR and in‐hospital outcomes.

**Methods:**

Data from the Improving Care for Cardiovascular Disease in China‐AF project from 2014 to 2019 were retrospectively analyzed. The primary outcome was the composite of in‐hospital all‐cause mortality and in‐hospital acute heart failure (AHF). Secondary outcomes included stroke/transient ischemic attack (TIA) and bleeding during hospitalization. Logistic regression analyses were used to assess the association between RHR and outcomes.

**Results:**

Our study included 12,775 patients hospitalized for AF in 236 hospitals. Logistic regression analyses using different models showed a significant association between RHR exceeding 80 bpm and an increased risk of the primary outcome (adjusted OR: 1.79 [95% CI: 1.44–2.22]). A positive association between RHR and the primary outcome was identified with RHR ≥ 80 bpm. Marginal effect analyses showed that patients with advanced AF types were at higher risk across the range of RHR. Conversely, catheter ablation, but not antiarrhythmic drug use, was associated with a decreased risk.

**Conclusion:**

A significant association was identified between RHR and adverse outcomes in patients hospitalized for AF, where RHR exceeding 80 bpm was associated with an increased risk.

**Trial Registration:** ClinicalTrials.gov identifier: NCT02309398

## 1. Introduction

The management of atrial fibrillation (AF) extends beyond the traditional dichotomy of rate versus rhythm control. Previously, when focusing on the selection of either rate or rhythm control, there was limited clarity on the target of heart rate. However, most patients today benefit from a tailored combination of both strategies, making the optimal heart rate target even more uncertain in real‐world scenarios [1, 2].

Hospitalized patients with AF often face an elevated risk of complications, including stroke, exacerbation of heart failure (HF), and haemodynamic instability. These patients were mainly managed with rate control therapies [3]. Identifying the relationship between resting heart rate (RHR) and in‐hospital outcomes may significantly contribute to reducing adverse events, improving prognosis, and providing valuable guidance for the management of hospitalized patients with AF or those with worsening symptoms. In this study, we sought to determine the dose–response relationships between RHR and adverse outcomes in patients hospitalized for AF using a large sample database from the Improving Care for Cardiovascular Disease in China‐AF (CCC‐AF) project.

## 2. Methods

**Figure 1:**
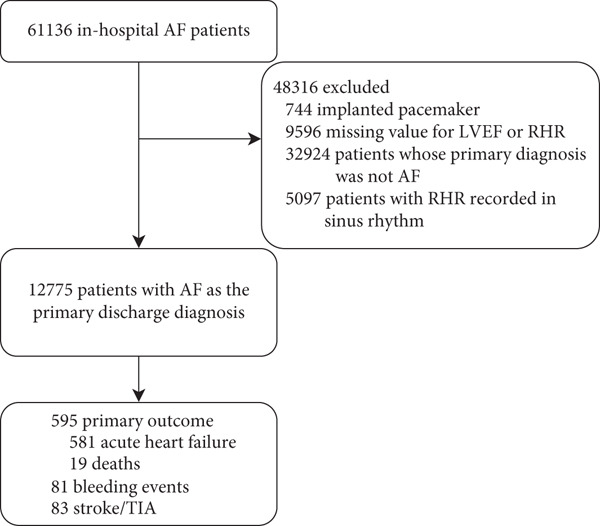
Study flow chart. AF, atrial fibrillation; LVEF, left ventricular ejection fraction; RHR, resting heart rate; TIA, transient ischemic attack.

The CCC‐AF project is a nationwide quality improvement initiative in China, launched in 2014 as a collaboration between the American Heart Association (AHA) and the Chinese Society of Cardiology (CSC). It involved a total of 236 hospitals, including 151 tertiary hospitals and 85 secondary hospitals from seven regions of China. The design and methodology of the CCC‐AF project have been meticulously documented and published [4]. In each hospital, the first 10–20 hospitalized patients with AF were retrospectively enrolled in a consecutive manner each month. This project consists of a project management group and a senior management group (SMG). Clinical volunteers of the SMG communicate from AHA and CSC frequently via teleconferences, emails, and face‐to‐face meetings to ensure the scientific integrity and supervise the implementation of the CCC‐AF project. Clinical data elements were collected according to the recommendations on the data standards for clinical research in AF [5, 6]. Data collection and analysis are managed by the daily routine management group and the data group, with guidance provided by the SMG. This project complied with the Declaration of Helsinki. The CCC‐AF project was approved by the institutional review board of Beijing Anzhen Hospital with a waiver of informed consent. Clinical data of 61,136 inpatients diagnosed with AF were collected from January 2015 to December 2019. To minimize potential bias and to focus on the risks and outcomes associated with AF, this study included only patients whose primary diagnosis was AF, as documented in the hospital information system. RHR and heart rhythm were recorded using the first 12‐lead electrocardiogram (ECG) following 5 min of rest in the hospital records and prior to getting in‐hospital treatment. In this study, only patients whose admission ECG demonstrated AF were included while patients with RHR recorded in sinus rhythm were excluded. The inclusion and exclusion criteria are shown in Figure 1. The final sample consisted of 12,775 patients with AF.

Study variables included the types of AF (first diagnosed AF, paroxysmal AF, persistent AF, and long‐standing persistent/permanent AF), valvular AF, demographic information (age and sex), medical history (smoking, drinking, hypertension, diabetes, coronary artery disease, HF, and cerebrovascular disease), clinical variables (systolic blood pressure, RHR, estimated glomerular filtration rate [eGFR], and left ventricular ejection fraction [LVEF]), prehospital treatment (anticoagulant, calcium channel blocker, *β* blocker, other antiarrhythmic drug [AAD], and angiotensin‐converting enzyme inhibitor/angiotensin receptor blocker [ACEI/ARB]), and in‐hospital treatment (anticoagulant, calcium channel blocker, *β* blocker, other AAD, and catheter ablation). The eGFR was calculated according to the Chronic Kidney Disease Epidemiology Collaboration equation [7]. Other AADs comprise amiodarone, flecainide, propafenone, dofetilide, dronedarone, and ibutilide. Patients’ symptoms were assessed by the European Heart Rhythm Association (EHRA) symptom classes (I–IV), where Class I means asymptomatic [8].

Because heart rate is directly related to HF, the primary outcome was the composite of new‐onset acute HF (AHF) and all‐cause mortality during hospitalization. The diagnosis of AHF was based on the guidelines, characterizing it as a new onset or exacerbation of symptoms and signs indicative of HF. The secondary outcomes included in‐hospital stroke/transient ischemic attack (TIA) and bleeding. Missing values of eGFR (4.97%) and systolic blood pressure (0.87%) were imputed using sequential regression multiple imputation with IVEware Version 0.2 (Survey Research Center, University of Michigan, Ann Arbor, Michigan, United States).

Continuous variables are summarized as mean ± standard deviation or median (interquartile range), and comparisons were performed using the *t*‐test or Mann–Whitney *U* test according to the distribution. Categorical variables are expressed as the number (percentage) for which the chi‐square test was used.

The RHR was categorized into two groups based on the RACE II trial and guidelines: < 80 bpm and ≥ 80 bpm. The association between RHR and the primary outcome was estimated with various logistic regression models by computing the odds ratio (OR) and 95% confidence interval (CI): (1) Model 1: univariable regression model; (2) Model 2: multivariable regression model adjusted by sex, age, smoking, drinking, hypertension, diabetes, coronary artery disease, HF, cerebrovascular disease, types of AF, valvular AF, asymptomatic AF, systolic blood pressure, eGFR, and LVEF; and (3) Model 3: adjusted by the covariates in Model 2 and additionally prehospital and in‐hospital treatment. The adjusted absolute risk difference compared to the reference RHR (< 80 bpm) was calculated.

The relationship between RHR and outcomes was further assessed using the restricted cubic spline (RCS) within a fully adjusted logistic regression model (Model 3). The reference value (73 bpm) was set at the point with the lowest risk of the primary outcome, with all other adjusted variables set at their median values (for continuous variables) or reference values (for categorical variables). The number of knots (five knots for the primary outcome and three knots for the secondary outcomes) was selected based on the value associated with the lowest Akaike information criterion in the model. RHR was then categorized into nine distinct categories spanning from 0 to 220 bpm. This categorization is aimed at evaluating its relationship with the outcomes using the floating risk method based on the Model 3, with a reference RHR set at 60–80 bpm. This facilitates comparisons among various exposure categories, eliminating the need to select an arbitrary baseline group for displaying CIs. Marginal effect analyses based on the Model 3 of logistic regression were used to illustrate the impact of different exposures on the relationship between RHR and the primary outcome [9]. The RHR spectrum was manually set from 20 to 220 bpm, with intervals of 5 bpm.

The statistical analyses were performed using R Version 4.2.2 (The R Project for Statistical Computing, Vienna, Austria) and STATA Version 17 (Stata Corp., College Station, Texas, United States). A two‐sided *p* < 0.05 was considered statistically significant.

## 3. Results

A total of 12,775 patients were enrolled with a median hospitalization of 8 (6–11) days. Baseline characteristics of the participants are summarized in Table 1. Patients with RHR < 80 bpm were older and more likely to be male. These patients exhibited elevated systolic blood pressure, higher LVEF, and increased prevalence of hypertension and cerebrovascular disease. However, they have lower eGFR and lower incidence of HF. Considering prehospital treatment, patients with RHR < 80 bpm had a higher rate of anticoagulant intake. During hospitalization, these patients had a lower rate of *β* blocker and other AAD use, but a higher proportion of catheter ablation.

**Table 1:** Baseline characteristics of the study population according to the RHR.

**Variables**		**RHR category**		**p** **value**
	**Total (** **n** = 12,775**)**	**R** **H** **R** < 80 **(** **n** = 4298**)**	**R** **H** **R** ≥ 80 **(** **n** = 8477**)**	
Age (years)	66.90 ± 11.97	67.86 ± 11.74	66.41 ± 12.06	< 0.001
Male sex, *n* (%)	7234 (56.63)	2603 (60.56)	4631 (54.63)	< 0.001
Previous history, *n* (%)				
Smoking	2711 (21.22)	920 (21.41)	1791 (21.13)	0.734
Drinking	1718 (13.45)	577 (13.42)	1141 (13.46)	0.978
Hypertension	6703 (52.47)	2325 (54.09)	4378 (51.65)	0.009
Diabetes	1894 (14.83)	638 (14.84)	1256 (14.82)	0.988
Coronary artery disease	1915 (14.99)	671 (15.61)	1244 (14.68)	0.169
Heart failure	806 (6.31)	213 (4.96)	593 (7.00)	< 0.001
Cerebrovascular disease	1687 (13.21)	629 (14.63)	1058 (12.48)	0.001
Valvular AF, *n* (%)	1028 (8.05)	354 (8.24)	674 (7.95)	0.599
EHRA class, *n* (%)				
I	774 (6.06%)	287 (6.68)	487 (5.74)	< 0.001
II	7167 (56.10)	2527 (58.79)	4640 (54.74)	
III	4439 (34.75)	1381 (32.13)	3058 (36.07)	
IV	395 (3.09)	103 (2.40)	292 (3.44)	
Types of AF, *n* (%)				
First diagnosed	2692 (21.07)	758 (17.64)	1934 (22.81)	< 0.001
Paroxysmal	4695 (36.75)	1586 (36.90)	3109 (36.68)	
Persistent	4052 (31.72)	1447 (33.67)	2605 (30.73)	
Long‐standing persistent/permanent	1336 (10.46)	507 (11.80)	829 (9.78)	
Systolic blood pressure (mmHg)	129.92 ± 19.40	131.39 ± 19.61	129.17 ± 19.25	< 0.001
Resting heart rate (bpm)	95.12 ± 29.06	66.48 ± 9.54	109.63 ± 24.49	< 0.001
eGFR (mL/min/1.73 m^2^)	79.34 ± 22.26	78.44 ± 22.19	79.80 ± 22.28	0.001
LVEF (%)	58.86 ± 9.62	60.47 ± 8.68	58.04 ± 9.97	< 0.001
Prehospital treatment, *n* (%)				
Anticoagulant	2858 (22.37)	1100 (25.59)	1758 (20.74)	< 0.001
Calcium channel blocker	1823 (14.27)	628 (14.61)	1195 (14.10)	0.448
*β* blocker	3707 (29.02)	1257 (29.25)	2450 (28.90)	0.700
Other AAD	1367 (10.70)	488 (11.35)	879 (10.39)	0.095
ACEI/ARB	2090 (16.36)	721 (16.78)	1369 (16.15)	0.380
In‐hospital treatment, *n* (%)				
Anticoagulant	8695 (68.06)	2899 (67.45)	5796 (68.37)	0.300
Calcium channel blocker	2220 (17.38)	774 (18.01)	1446 (17.06)	0.189
*β* blocker	7219 (56.51)	1919 (44.65)	5300 (62.52)	< 0.001
Other AAD	4228 (33.10)	1328 (30.90)	2900 (34.21)	< 0.001
Catheter ablation	3181 (24.90)	1192 (27.73)	1989 (23.46)	< 0.001

Abbreviations: AAD, antiarrhythmic drug; ACEI, angiotensin‐converting enzyme inhibitor; AF, atrial fibrillation; ARB, angiotensin receptor blocker; eGFR, estimated glomerular filtration rate; EHRA, European Heart Rhythm Association; LVEF, left ventricular ejection fraction; RHR, resting heart rate.

Patients with RHR ≥ 80 bpm exhibit an elevated risk of the primary outcome in all logistic regression models, mainly driven by the associations with AHF (Table 2). In the fully adjusted model, RHR ≥ 80 bpm was associated with an OR of 1.79 (95% CI: 1.44–2.22, *p* < 0.001), accounting for a 2.11% (95% CI: 1.40%–2.83%) increase in absolute risk. On a per‐event basis, RHR ≥ 80 bpm was associated with an increased risk of AHF in all logistic models. In the fully adjusted model, RHR ≥ 80 bpm was associated with an OR of 1.77 (95% CI: 1.42–2.20, *p* < 0.001) for AHF, corresponding to an absolute risk increase of 2.03% (95% CI: 1.33%–2.74%, *p* < 0.001) for AHF.

**Table 2:** Univariable and multivariable logistic regression analyses according to the RHR.

	**RHR category (< 80 or ≥ 80 bpm)**			
	**OR (95% CI)**	**p** **value**	**Absolute risk difference (95% CI)**	**p** **value**
Model 1				
Primary outcome	1.98 (1.62–2.42)	< 0.001	2.67% (1.97%–3.37%)	< 0.001
Heart failure	1.98 (1.62–2.43)	< 0.001	2.61% (1.92%–3.30%)	< 0.001
All‐cause mortality	1.90 (0.63–5.74)	0.253	0.08% (−0.04% to 0.21%)	0.198
Stroke/TIA	1.11 (0.70–1.77)	0.654	0.07% (−0.22% to 0.36%)	0.648
Bleeding events	1.01 (0.64–1.61)	0.953	0.01% (−0.28% to 0.30%)	0.953
Model 2				
Primary outcome	1.85 (1.50–2.29)	< 0.001	2.24% (1.54%–2.95%)	< 0.001
Heart failure	1.84 (1.48–2.28)	< 0.001	2.18% (1.48%–2.87%)	< 0.001
All‐cause mortality	2.03 (0.66–6.28)	0.219	0.11% (−0.05% to 0.27%)	0.166
Stroke/TIA	1.10 (0.68–1.77)	0.689	0.06% (−0.23% to 0.36%)	0.684
Bleeding events	1.03 (0.64–1.65)	0.899	0.02% (−0.27% to 0.31%)	0.898
Model 3				
Primary outcome	1.79 (1.44–2.22)	< 0.001	2.11% (1.40%–2.83%)	< 0.001
Heart failure	1.77 (1.42–2.20)	< 0.001	2.03% (1.33%–2.74%)	< 0.001
All‐cause mortality	2.40 (0.76–7.60)	0.138	0.13% (−0.02% to 0.29%)	0.094
Stroke/TIA	1.18 (0.73–1.93)	0.497	0.10% (−0.19% to 0.40%)	0.486
Bleeding events	1.17 (0.72–1.89)	0.526	0.10% (−0.19% to 0.38%)	0.517

*Note:* Model 1: unadjusted. Model 2: adjusted for covariates (without treatment) listed in the Methods section. Model 3: adjusted for covariates listed in the Methods section.

Abbreviations: RHR, resting heart rate; TIA, transient ischemic attack.

**Figure 3:**
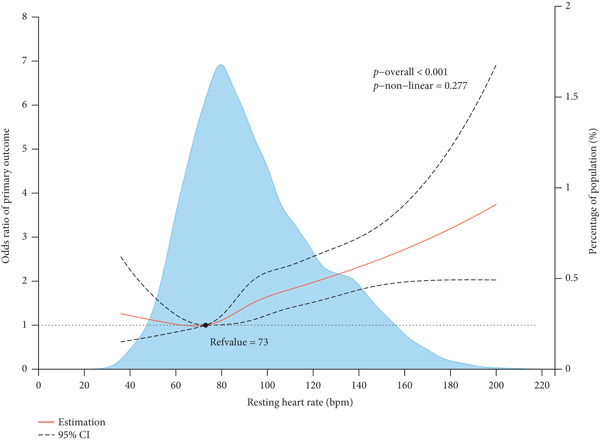
Restricted cubic spline for the primary outcome in the fully adjusted logistic regression model. The reference point was set at the lowest risk for the primary outcome (resting heart rate at 73 bpm). The background density plot (light blue) represents the density distribution of RHR in the study population. The red central lines represent the estimated adjusted odds ratio, and the black dashed lines represent the 95% confidence interval.

The incidence and fully adjusted risk of the primary outcome gradually increased with RHR categories ≥ 80 bpm (Figure 2). A positive association was observed with increased risk of RHR categories ≥ 80 bpm (Figure 2b). Further analysis using RCS with five knots in the fully adjusted logistic regression model revealed a general U‐shaped relationship between RHR and the primary outcome. Specifically, the increased risk of the primary outcome was observed when RHR > 73 bpm (Figure 3).

Figure 2The primary outcome according to resting heart rate categories. (a) Incidence of the primary outcome during hospitalization according to resting heart rate categories. (b) Fully adjusted odds ratio and 95% confidence intervals in each category using the floating risk method.

**Figure:**
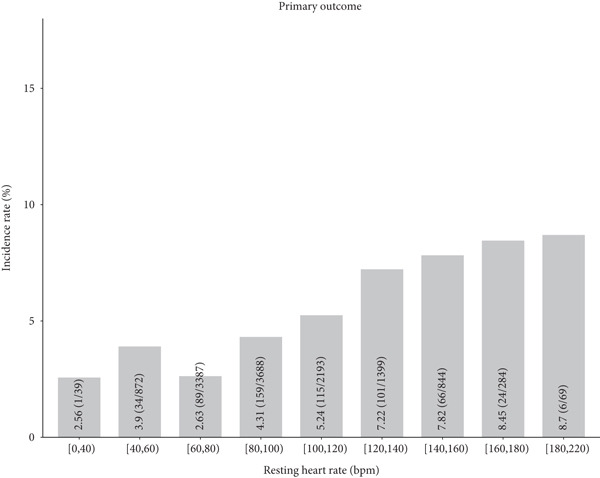
(a)

**Figure:**
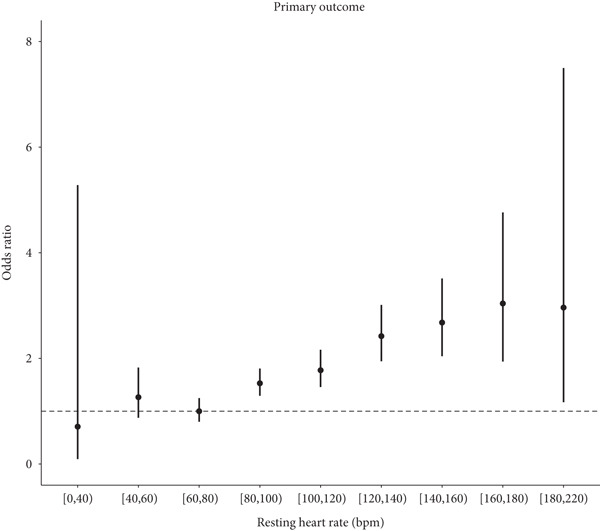
(b)

**Figure 4:**
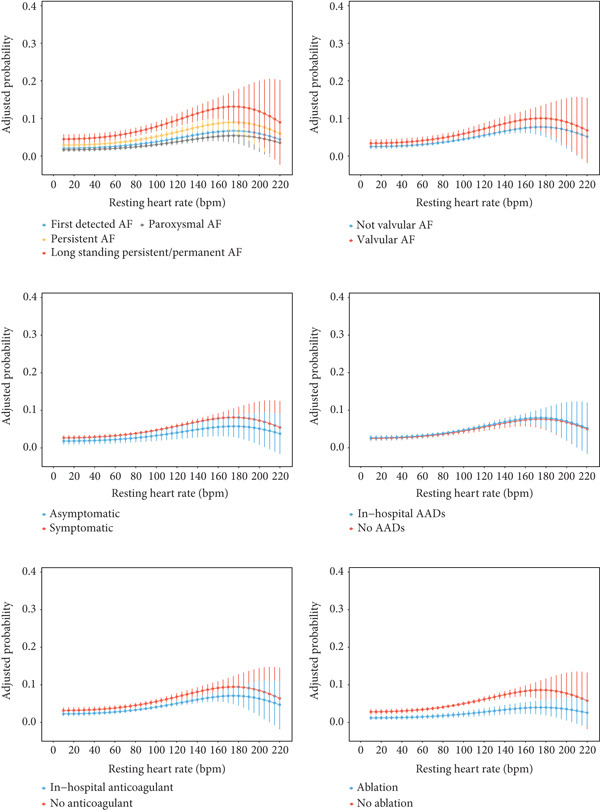
Marginal effects of different exposures on the risk of the primary outcome across the full range of resting heart rate in the fully adjusted logistic regression model. The vertical lines indicate 95% confidence intervals. AF, atrial fibrillation.

Figure 4 displays how different exposures were associated with the dose–response relationship across the RHR range. Patients with more advanced AF types were associated with an increased risk of the primary outcome across the RHR spectrum. Conversely, the use of catheter ablation was associated with a lower risk of the primary outcome.

## 4. Discussion

In this nationwide AF registry with a large sample size, we revealed a significant association between RHR and the primary outcome, with higher risks for high RHR after controlling for baseline covariates. In particular, our analysis revealed an increased risk in patients with an RHR exceeding 80 bpm.

Although early rhythm control therapy has gradually become the leading strategy for AF, the use of AADs without catheter ablation is not superior to the rate control [10–12]. In fact, all AADs have the effect of rate control, and most rhythm control therapies are under the background of rate control. Therefore, rate control is still the cornerstone for the management of AF, especially in older patients with a long history of AF and a larger left atrium [2]. The scientific evidence for heart rate control in hospitalized patients with AF is limited, with previous recommendations focusing primarily on long‐term follow‐up. In recent years, guidelines have increasingly advocated the use of lenient rate control in the management of AF. Before the RACE II trial, the guideline empirically recommended an RHR of 60–80 bpm as the target [13]. Subsequently, based on the RACE II trial, the ESC guideline of the same year recommended lenient control (< 110 bpm) for those with no or mild symptoms. Similarly, the 2014 ACC guideline recommended a strict rate control strategy (< 80 bpm) for symptomatic patients. In contrast, the recent guideline recommends lenient control (< 100–110 bpm) for those without HF and with no or mild symptoms [2].

Many studies suggest that elevated heart rate is a risk factor for adverse outcomes. Setting the target for RHR control at an even lower value may reveal more pronounced benefits compared to lenient rate control. In patients with sinus rhythm or AF, a heart rate ≥ 75 bpm was associated with higher mortality and all‐cause readmission [14]. Higher admission heart rate is independently associated with worse in‐hospital outcomes in patients hospitalized for HF (in sinus rhythm or AF), with the lowest mortality rate associated with heart rate between 70 and 75 [15]. In patients with AF but no HF, increased heart rate was independently associated with HF [16]. In addition, RHR > 80 bpm was associated with an increased risk of stroke [17, 18]. In patients with chronic HF, RHR ≥ 87 bpm was associated with more than double the risk of HF worsening and mortality compared with those with 70–72 bpm [19]. A study showed that RHR higher than 100 bpm was associated with an increased risk of HF and all‐cause mortality [20]. Notably, the RCS curve indicated that the risk of HF and mortality begins to rise at an RHR above 80 bpm. Recently, the longitudinal evaluation of RHR with a median of 3.4 years showed that RHR higher than 80 bpm was associated with an increased risk of mortality in patients with AF [21].

Strict rate control may be particularly important in patients with more progressive AF types. We showed a positive correlation between AF types and risk of the primary outcome, with long‐standing persistent/permanent AF having the highest risk. This is consistent with the previous study where patients with nonparoxysmal AF and elevated RHR had an increased risk of adverse cardiac events [16, 22]. RHR > 80 bpm and the presence of HF were strongly associated with progression of AF to a more persistent form [23].

In recent studies, catheter ablation for AF in patients with HF has been associated with improved cardiac function, quality of life, and significantly lower rates of composite outcomes, including all‐cause mortality and hospitalization for worsening HF [24–26]. Similarly, catheter ablation has been associated with reduced risk of hospitalization and mortality compared with AAD use in patients with HF and persistent AF [27]. In this study, we showed that catheter ablation, but not AAD, was associated with decreased risk of the primary outcome across the entire RHR spectrum. We acknowledge that the benefit of catheter ablation may be confounded by selection bias (e.g., healthier patients may undergo ablation). To mitigate this concern, we adjusted many covariates to detect the independent effect. However, other confounding by unmeasured or imperfectly captured factors such as socioeconomic status, medication dosage, and patient frailty remains a concern.

Although the study used only one baseline RHR, it can still provide insight into RHR over a short period of time. A previous study using the largest database of RHR demonstrated that the individual fluctuation of RHR during a single week was only 3 bpm. In approximately 80% of individuals, the maximum weekly fluctuation was less than 10 bpm. Taking into account diurnal variability, there was an approximate difference of 4 bpm in RHR between day and night [28, 29]. It is worth noting that the mean RHR for the overall population was approximately 66 bpm, which is consistent with the RHR we observed to be associated with the lowest risk [29].

### 4.1. Study Limitations

First, this observational study demonstrates an association between RHR and in‐hospital outcomes but cannot establish causation; its findings are therefore hypothesis‐generating. Whether actively targeting specific in‐hospital RHR values improves outcomes remains unknown due to the lack of interventional evidence. Further randomized controlled trials with strict grouping of patients according to heart rate targets are needed. Second, we did not have data on the levels of HF‐specific biomarkers such as N‐terminal pro‐B‐type natriuretic peptide in our study population. Third, we showed that catheter ablation was associated with decreased risk of the primary outcome. However, whether this effect was directly related to influences in RHR is unknown since we have only the admission RHR value. Fourth, information regarding medication dosage was not collected in the CCC‐AF project. Future studies incorporating detailed medication dosage would be valuable to better understand the impact of different rate control strategies on clinical outcomes. Fifth, haemodynamic instability during acute hospitalization often leads to substantial RHR fluctuations. A single timepoint assessment therefore fails to capture the chronic baseline RHR reliably, thereby introducing potential misclassification and precluding analysis of longitudinal RHR patterns in relation to outcomes. Sixth, only 19 deaths occurred among 12,775 patients, resulting in a low event rate (0.16%) that renders these estimates statistically underpowered; consequently, these mortality data should be viewed as exploratory and hypothesis‐generating.

## 5. Conclusions

Data from the national registry revealed a positive association between RHR and the adverse composite outcome of HF and all‐cause mortality in hospitalized patients with AF, with increased risk at high RHR.

**Nomenclature**
AAD: antiarrhythmic drug
ACEI/ARB: angiotensin‐converting enzyme inhibitor/angiotensin receptor blocker
AF: atrial fibrillation
AHF: acute heart failure
CCC‐AF: Improving Care for Cardiovascular Disease in China‐AF
CSC: Chinese Society of Cardiology
ECG: electrocardiogram
eGFR: estimated glomerular filtration rate
EHRA: European Heart Rhythm Association
HF: heart failure
LVEF: left ventricular ejection fraction
RCS: restricted cubic spline
RHR: resting heart rate
SMG: senior management group
TIA: transient ischemic attack

## Disclosure

All authors contributed to the final approval of the version to be published. All authors agreed to be accountable for all aspects of the work.

## Conflicts of Interest

The authors declare no conflicts of interest.

## Author Contributions

Kangning Han, Deyong Long, and Changsheng Ma were involved in the conception and design of the study. Kangning Han, Xia Li, and Na Yang performed the statistical analysis. Kangning Han, Xia Li, Jing Liu, Na Yang, Yongchen Hao, and Dong Zhao interpreted the data. Kangning Han and Xia Li drafted the manuscript. Biao Fu, Mengmeng Li, Tong Liu, Ying Peng, Chenxi Jiang, Ribo Tang, Jianzeng Dong, Dong Zhao, Deyong Long, and Changsheng Ma revised the manuscript. All authors contributed to the acquisition of data.

## Funding

The study was funded by the National Natural Science Foundation of China (82151306) and Noncommunicable Chronic Diseases—National Science and Technology Major Project (2023ZD0504200).

## Data Availability

Data are available from the authors upon reasonable request and with permission of Beijing Anzhen Hospital, Capital Medical University.
